# Atmospheric drought and low light impede mycorrhizal effects on leaf photosynthesis—a glasshouse study on tomato under naturally fluctuating environmental conditions

**DOI:** 10.1007/s00572-018-0872-6

**Published:** 2018-10-31

**Authors:** Michael Bitterlich, Philipp Franken, Jan Graefe

**Affiliations:** Leibniz-Institute of Vegetable and Ornamental Crops e.V., Theodor-Echtermeyer-Weg 1, 14979 Großbeeren, Germany

**Keywords:** Arbuscular mycorrhiza, Light, Humidity, Photosynthesis, Stomatal conductance

## Abstract

Arbuscular mycorrhiza fungi (AMF) consume plant carbon and impact photosynthesis, but effects of AMF on plant gas exchange are transient and hardly predictable. This is at least partially because plant-internal nutrient-, water-, and sink-related effects, which can be influenced AMF, and atmospheric conditions integrate at the photosynthesis level. In nature and in plant production, plants face periodical and random short-term switches of environmental conditions that limit photosynthesis, which may impede stimulatory effects of AMF on leaf photosynthetic capacities. We hypothesized that mycorrhizal effects on plant internal-photosynthetic potentials will only translate to actual photosynthetic rates, if atmospheric conditions do not superimpose limitations to the photosynthetic process. We aimed to cover wide ranges of within and between-day variations in light intensities and vapor pressure deficits with an untargeted approach. We grew tomato plants hydroponically for 8 weeks in open pots and irrigated beyond pot water capacity every morning. Plants were inoculated or not with *Funneliformis mosseae* and were fertilized with a low-strength nutrient solution, which guaranteed good AMF colonization and comparable sets of mycorrhizal and non-mycorrhizal plants regarding developmental stage and leaf age. Instantaneous leaf photosynthesis was monitored continuously with transparent chambers during 3 days under naturally fluctuating greenhouse conditions on the two uppermost fully expanded leaves. We fitted mechanistic gas exchange models and modeled continuous daytime dynamics of net photosynthetic rates and stomatal conductance for representative sunlit canopies of random populations of mycorrhizal and non-mycorrhizal plants. Depending on time, mycorrhizal plants showed enhanced or decreased stomatal conductance over wide ranges of light intensities. Higher or lower stomatal opening in mycorrhizal plants became ineffective for photosynthetic rates under low light. In contrast and in accordance with the effects on stomatal conductance, photosynthetic rates were comparatively increased or decreased in mycorrhizal plants under high light conditions. This required at least moderate vapor pressure deficits. Under high atmospheric drought, stomatal conductance strongly declined in all plants, which also capped maximum photosynthetic rates under high light. Leaf photosynthetic capacities were higher in mycorrhizal plants when leaves contained more proteins and/or the plant-internal moisture stress was lower than in non-mycorrhizal plants. However, this only resulted in enhanced photosynthetic rates as long as leaves were not exposed to low radiation or high atmospheric drought. We conclude that light and atmospheric moisture are decisive factors for potential carbon cost and gain scenarios of plants associated with AMF.

## Introduction

In search for sustainable plant production, arbuscular mycorrhizal fungi (AMF) have become an attractive target for implementation as biostimulants (Rouphael et al. 2015). The biostimulant-potential of AMF is based on observations that, among other effects, AMF can contribute to plant uptake of mineral nutrients (Marschner and Dell 1994; Nouri et al. 2014) and facilitate plant drought tolerance (e.g., Ruiz-Lozano et al. 1995; Porcel and Ruiz-Lozano 2004). Symbioses of AMF with plants is characterized by bi-directional cross-kingdom transfers of phosphorus (P), nitrogen (N), and compounds rich in carbon (C) (Govindarajulu et al. 2005; Helber et al. 2011; Keymer et al. 2017; Rausch et al. 2001). In addition, AMF can indirectly contribute to plant growth, nutrient uptake and drought tolerance by influencing the physico-chemical environment in the rooting zone that sets constraints to nutrient and water extractability (Leifheit et al. 2014; Rillig et al. 2010; Rillig and Mummey 2006; Bitterlich et al. 2018a, b). Both direct and indirect contributions to nutrient and water extractability from soils require that hyphae proliferate beyond the ambit of roots which are developed with sustenance by plant C fixed in photosynthesis (Smith and Read 2008). Hence, mycorrhizal plants possess an additional and significant C sink (as reviewed in: Řezáčová et al. 2017, 2018), which can be compensated by photosynthesis, provided that AMF do not only substitute other plant C sinks in the symbiotic interaction (Kaschuk et al. 2009; Řezáčová et al. 2018). Indeed, rates of photosynthesis of mycorrhizal plants are commonly altered in comparative studies with non-mycorrhizal (NM) counterparts (Augé 2001; Augé et al. 2014, 2016; Boldt et al. 2011).

Internal to leaves, photosynthesis is limited by diffusive and biochemical processes. CO_2_ diffusion through stomata to sites of C fixation limits CO_2_ availability, particularly in C_3_ species (e.g., Grassi and Magnani 2005). Downstream to CO_2_ diffusion, photosynthesis is limited biochemically by electron transport rate through the thylakoid membrane, carboxylation velocity in the Calvin cycle and the rate of end-product use (Von Caemmerer 2000). The capacities of these biochemical processes set the plant-internal limit to photosynthetic rates under light and/or CO_2_ saturation (Von Caemmerer 2000). All these plant-internal limitations to leaf photosynthesis are potentially influenced in plants when they are colonized by AMF.

Stomatal conductance often increases in mycorrhizal plants (Augé et al. 2014). Plants regulate stomatal aperture under drought to avoid excessive water loss and wilting (Dewar 2002; Tardieu and Davies 1993; Tardieu and Simonneau 1998) which limits CO_2_ influx. Modulations of drought responses and stomatal conductance in mycorrhizal plants are thought to relate to P nutrition, to adjustments of root hydraulic conductivities and aquaporin expression, to altered ABA production and signaling, to hyphal water transport and/or to changes of the hydraulic properties in the growth substrate (see, e.g., Allen et al. 1981; Augé 2001, 2004; Augé et al. 1995; Aroca et al. 2007; Bitterlich et al. 2018b; Duan et al. 1996; Ebel et al. 1997; Nelsen and Safir 1982; Ruiz-Lozano et al. 1995, 2009; Ruth et al. 2011).

Leaf N and, in particular, P levels are frequently affected in mycorrhizal plants and, hence, leaf N and P levels have been observed to scale equally with photosynthesis in mycorrhizal and NM plants (Black et al. 2000; Grimoldi et al. 2006). This is because capacities of biochemical processes in chloroplasts (electron transport capacity through the thylakoid membrane and carboxylation capacity in the Calvin cycle) are limited by tissue concentrations of N and P (e.g., Braune et al. 2009; Walker et al. 2014). Major parts of leaf N are found in chloroplast proteins like RuBisCO (Evans 1989) and P is conserved in ATP pools and reduction equivalents contributing to photophosphorylation and electron transport in chloroplasts (Jacob and Lawlor 1992, 1993). Downstream to electron transport and C fixation in chloroplasts, end-product limitation of photosynthesis (Paul and Foyer 2001) may also be alleviated in leaves by symbiotic sinks like rhizobia and/or AMF (Kaschuk et al. 2009, 2012; Wright et al. 1998) if increased sink strength causes enhanced rates of end-product use (triose-phosphate use) (Kaschuk et al. 2009, 2012).

Still, effects of AMF on actual photosynthetic rates are considered transient and hardly predictable; measured rates of stomatal conductance and photosynthesis are frequently not consistent with the growth outcome (Augé et al. 2014). At least partially, this is because potential drought-, nutrient-, or sink-related effects on photosynthetic processes induced by AMF may co-occur and can act synergistically or compensatorily. In addition, potential photosynthetic rates could be constantly higher in mycorrhizal plants as long as AMF facilitates tissue nutrient accumulation, alleviates plant moisture stress, or stimulates sink metabolism, but atmospheric conditions may superimpose restrictions to photsynthetic rates that cannot be entirely overcome by effects of AMF colonization on plant properties. For example, under low light, photosynthesis is mainly limited by incoming energy and, hence, by the rate of electron transport, but not directly by its capacity (Ögren and Evans 1993). And, stomata also close upon atmospheric drought, which limits CO_2_ availability at the sites of carboxylation (Merilo et al. 2018). Such atmospheric limitations may reduce the compensatory power of photosynthesis for the additional C costs of AMF and may be decisive whether or not and how strongly photosynthetic rates change in leaves of mycorrhizal plants.

To increase the predictability of gas exchange of mycorrhizal plants and to appropriately address the integrated nature of atmospheric and plant-internal limitations to photosynthetic processes, one would need (i) a framework that quantifies the plant-internal capacity of photosynthesis, which can be influenced by AMF and (ii) a monitoring of leaf gas exchange rates under variable atmospheric conditions that covers expected short-term switches between atmospheric and plant-internal limitations to photosynthesis.

We hypothesized that in an established symbiosis, AMF will affect the plant-internal photosynthetic capacity, but that this will only translate to changes in leaf photosynthetic rates if atmospheric conditions do not superimpose limitations by light and/or humidity. We used mechanistic gas exchange models to quantify the plant-internal nutrient and water limitations to photosynthesis (e.g., Braune et al. 2009; Medlyn et al. 2011). These models contain intrinsic parameters that are physiologically interpretable as plant-internal traits irrespective of environmental conditions (Medlyn et al. 2011; Von Caemmerer 2000; Walker et al. 2014). For introduction to the parameters, please refer to the material and methods. We monitored daytime leaf gas exchange under naturally fluctuating atmospheric conditions (temperature, light, humidity) and modeled daytime responses of a representative leaf canopy separately for populations of mycorrhizal and NM plants. This allowed us to conclude under which atmospheric conditions mycorrhizal effects on plant-internal traits become effective for photosynthetic activity. We identify atmospheric conditions (in light intensity and atmospheric moisture) that could be targeted in experimentation and plant production under which leaf gas exchange of mycorrhizal plants becomes (i) more efficient than that of NM plants, a scenario in which plants may acquire additional C to feed AMF without being necessarily larger, and (ii) we elucidate which atmospheric conditions offset putative mycorrhizal effects on leaf-internal photosynthetic capacities, a scenario in which photosynthetic rates remain unchanged and, hence, limit the compensatory power of photosynthesis for additional C demands of AMF.

## Material and methods

### Plant growth and experimental conditions

The experimental set-up, irrigation, and fertilization were done in a way that allowed variability in environmental conditions and that induced expected, but untargeted variability in plant internal traits between mycorrhizal and NM plants.

We grew 100 tomato plants (*Solanum lycopersicum* cv. “Moneymaker”) randomized in 3.5-L open pots (one plant per pot) in the glass-house on a sand:vermiculite mixture (1:1 *v*/*v*; sand: grain size 0.2–1 mm, Euroquarz, Ottendorf-Okrilla, Germany; vermiculite: agra-vermiculite; Pullrhenen, Rhenen, Nehterlands). Fifty-three plants were inoculated with *Funnelliformis mosseae* (BEG 12, MycAgroLab, Breteniere, France). The inoculum was applied as 10% of the substrate volume and was provided as a mixture with clay and zeolite as carrier material. The 47 NM plants were mock-inoculated with the same amount of autoclaved inoculum and 200 mL of a filtrate of the inoculum (approx. 100 mL of inoculum were filtered with 200 mL deionized water through a Whatman filter with particle retention of 4–7 μm). Plants were irrigated with 400 mL of nutrient solution (De Kreij et al. 1997) every other day containing 10% of the standard phosphate to guarantee good colonization (40% of full strength; N, 10.32 mM; P, 0.07 mM, K, 5.5 mM, Mg, 1.2 mM, S, 1.65 mM, Ca, 2.75 mM, Fe, 0.02 mM, pH, 6.2, EC, 1.6 mS). Every morning, we irrigated with additional water until pot water capacity was achieved. Due to the porosity of the substrate, this might rarely induce hypoxia. In addition, the hydroponic system allowed us to minimize nutrient related growth responses, but to achieve substantial root colonization. By frequently providing readily available nutrients with a nutrient solution with a high N/P ratio, plants may starve from P and thus allow AMF colonization, but mycorrhizal effects contributing to plant P uptake by P mobilization beyond the root zone is minimized. This may not completely abolish mycorrhizal growth responses, but this guaranteed the absence of substantial differences in leaf age and plant flowering, which could potentially affect photosynthesis in the target leaves (Paul and Foyer 2001; Suzuki et al. 1987). All plants were approx. 1 m in height, possessed 16 to 18 leaves and 74.4% were flowering. No growth differences were detected between mycorrhizal and NM plants.

**Fig. 1:**
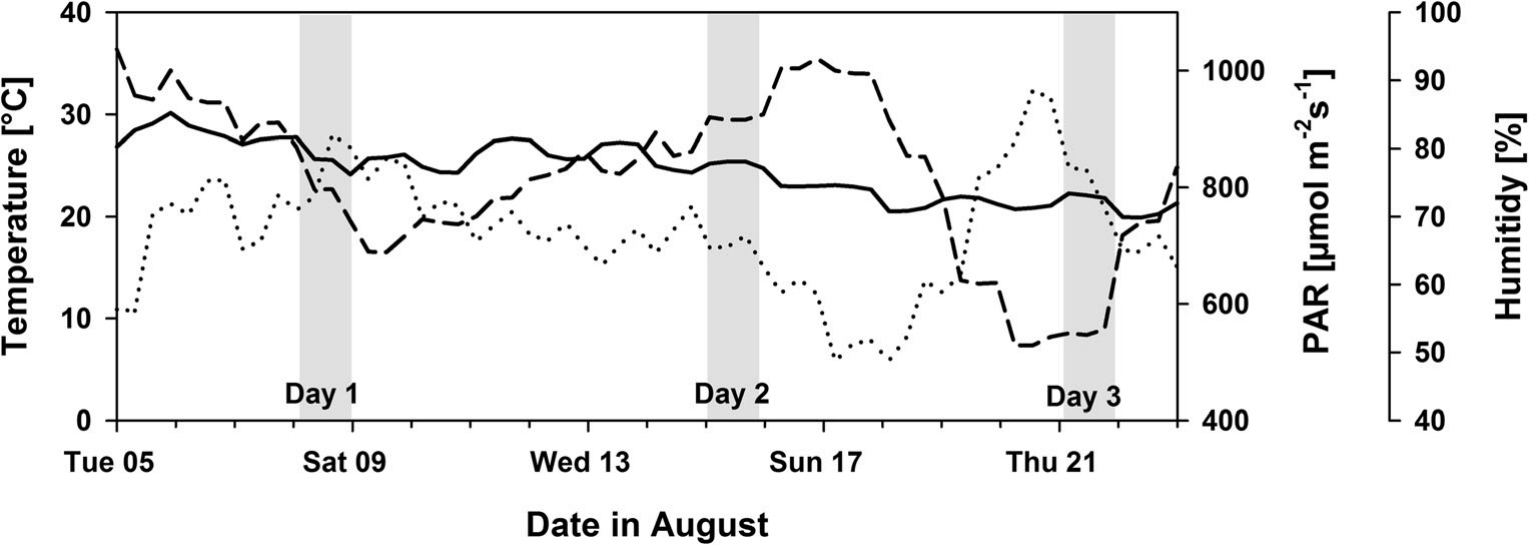
The mean temperature (solid line), photosynthetic active radiation (PAR; dashed line) and relative humidity (dotted line) in the glasshouse cabin during the experiment. Measurement days during the sixth (day 1), seventh (day 2), and eighth (day 3) weeks of growth are highlighted in gray

Plants were grown for 8 weeks during the summer from July to August in a glasshouse (52° 20′ 56.6′′ N 13° 18′ 36.7′′ E), only avoiding night temperatures below 15 °C (by heating) and intense heat (by ventilation). Figure 1 illustrates the atmospheric conditions from the sixth to the eighth week of the experiment. Within these weeks, measurements were carried out on three arbitrary days, as indicated.

While the daily mean temperatures remained below 25 °C and above 20 °C during most of the experiment, we observed marked changes in the mean irradiance (PAR) and relative humidity. We harvested 20 NM and 22 mycorrhizal plants after 6 weeks of growth (at the end of day 1) to check for growth responses and colonization. We harvested 27 NM and 31 mycorrhizal plants after 8 weeks for completion of all measurements (at the end of day 3).

The three measurement days were characterized by different atmospheric conditions at canopy height. The climatic conditions created evapotranspiration demands that were moderate at day 1 (mean *T* = 27 °C, mean PAR = 197 μmol m^−2^ s^−1^, min rH = 63%), high at day 2 (mean *T* = 26 °C, mean PAR = 370 μmol m^−2^ s^−1^, min rH = 38%), and moderate to low at day 3 (mean *T* = 24 °C, mean PAR = 201 μmol m^−2^ s^−1^, min rH = 50%).

### Leaf gas exchange measurements

In order to assess photosynthesis under a wide range of atmospheric factors, we measured leaf gas exchange continuously during daytimes under the conditions present in the glasshouse. This was done on the three arbitrary days during the sixth, seventh, and eighth week of growth.

Gas exchange measurements were carried out with two LI-6400XT portable photosynthesis systems (LICOR, Lincoln, USA) simultaneously on the two uppermost fully expanded leaves per plant. Transparent 6 cm^2^ chambers covered with Propafilm® were used that allowed maximum ambient light transmittance. The external quantum sensor for ambient irradiance was mounted in close proximity (≈ 3 cm away from the chamber). To alleviate fluctuations in [CO_2_] caused by the presence of operators and natural air ventilation, ambient glass-house air was drawn into the leaf chamber with a flow rate of 400 μmol s^−1^ from a buffer volume (200 L) in the middle of the glasshouse cabin. Ambient temperature in the glasshouse cabin was measured with a psychrometer and used to adjust leaf temperature in the chamber, which was computed via energy balance using the manufacturer’s instructions. Ambient relative humidity was measured with an open chamber before leaves were inserted and used as a control target with the inserted leaf. After switching leaves and achieving stable conditions in the chamber with inserted leaves (approx. 10 min in total), gas exchange activity was recorded 38 times with a logging interval of 15 s. Plants of different treatments were assessed alternately in quick succession. The measurements resulted in time courses of CO_2_ assimilation rates (A_n_) and stomatal conductance (g_sw_) under ambient fluctuating conditions, which resemble in particular light response curves of photosynthesis.

### Gas exchange models

We used gas exchange models (Farquhar et al. 1980; Medlyn et al. 2011) for two purposes: (i) to fit parameters that are closely related to plant internal traits, interpretable irrespective of environmental factors and (ii) to interpolate gas exchange rates at non-observed phases during the day.

The data have been used to fit parameters of the Farquhar model for C_3_ photosynthesis (Farquhar et al. 1980) according to Sharkey et al. (2007). The model is suitable to investigate the primary carbon fixation under changing conditions and contains parameters that are physiologically interpretable, irrespective of environmental factors. The model assumes that the CO_2_ assimilation rate (A_n_) is limited either by the carboxylation velocity in the Calvin cycle (RuBisCO-limited), by the rate of electron transport for ribulose-bisphosphate regeneration, or by the triose phosphate export rate and, is overall reduced by mitochondrial respiration (R_D_). The minimum of these rates determines measured A_n_. Under light saturation, photosynthesis is biochemically limited by carbon fixation in the Calvin cycle, which depends on substrate availability (CO_2_). If [CO_2_] at sites of carboxylation also approaches saturation under saturating irradiance, then photosynthesis is limited by triose phosphate use (A_T_, von Caemmerer 2000). Because the dataset did not provide sufficient variation in leaf internal [CO_2_] under light saturation to reliably assess RuBisCO-limited photosynthesis, we assumed the carboxylation capacity in the Calvin cycle (V_cmax_) required to compute A_C_ to be V_cmax_ = 0.5J_max_ as this ratio is commonly observed and conserved over C_3_ species (Wullschleger 1993). Similarly, assuming a hypothetical triose phosphate limited photosynthesis rate with *A*_*T*_ ≈ 0.5*V*_*cmax*_ ≈ 0.25*J*_*max*_ (Collatz et al. 1991) results in an ambient photosynthesis rate of about A_n_ = 30 μmol m^2^ s^−1^. As those values were never observed, we decided to omit triose phosphate transport limitation (*A*_*T*_) from the model fit. Thus, we assume the net photosynthetic rate defined as follows:

1 $$ {A}_n=\min\ \left\{{A}_J,{A}_C\right\}-{R}_D $$

where *A*_*J*_ is the electron transport-limited rate of CO_2_ assimilation and *A*_*C*_ is the RuBisCO-limited rate of CO_2_ assimilation. *A*_*J*_ is defined as follows:

2 $$ {A}_J=J\ \left[\frac{C_i-{\varGamma}^{\ast }}{4{C}_i+8{\varGamma}^{\ast }}\right] $$

where *J* is the rate of electron transport, *C*_*i*_ is the [CO_2_] at substomatal cavities and Γ^*^ is the CO_2_ compensation point in the absence of dark respiration. Equation (2) adopts limitations caused by NADPH supply, being one product of thylakoid electron transport used in regeneration of ribulose-bisphosphate in the Calvin cycle (Von Caemmerer 2000). Γ^*^ was determined after Laisk (1977) on an independent set of eight leaves and was 31.5 μmol mol^−1^ at 20 °C. The fitting of the light response of electron transport J (PAR) was performed with the non-rectangular hyperbola:

3 $$ J(PAR)=\frac{\ \alpha +{J}_{max}-\sqrt{{\left(\alpha\ x\  PAR+{J}_{max}\right)}^2-4\varTheta\ x\ \alpha\ x\  PAR\ x\ {J}_{max}}}{2\varTheta } $$

where PAR is the photosynthetic active radiation, α is the initial slope of electron transport at zero light (J(0)) and Θ is the curvature parameter (set to 0.51). Values for α ranged between 0.15 and 0.25. J_max_ is the maximum electron transport rate under light saturation. Hence, J_max_ is related to the leaf internal capacity of photosynthesis, which depends on chloroplast biochemistry and therefore on the leaf nutritional status. Thus, J_max_ is a target parameter for investigations that use treatments that affect the plant internal physiological state, such as inoculation with AM fungi.

Temperature response of parameters has been accounted for after Bernacchi et al. (2003):

4 $$ \mathrm{Parameter}={e}^{\left(\frac{\ c-\Delta  Ha}{R\left({T}_L+273\right)}\right)} $$

where Γ^*^ is the fitting parameter, T_L_ is the leaf temperature, c is a scaling constant, R is the molar gas constant (8.314 10^−3^ kJ K^−1^ mol^−1^) and ∆Ha is the activation energy. ∆Ha, and c have been used as given by Bernacchi et al. (2003). To allow across-day comparisons of J_max_ and R_D_ and relations to independent measured leaf traits, the parameters both have been scaled to 25 °C (J_max_^25^, R_D_^25^).

The Medlyn stomatal conductance model (Medlyn et al. 2011) was used to compute daytime dynamics of stomatal conductance and to fit a plant internal water use efficiency parameter. The model is based on the theory that stomata are regulated optimally to minimize water loss per unit carbon gained (Medlyn et al. 2011) and can be stated as follows:

5 $$ {g}_{sw}={g}_0+1.6\left(1+\frac{g_1}{\sqrt{VPD}}\right)\frac{A_n}{C_s} $$

where g_0_ is the leaf conductance of water vapor at zero or negative net photosynthesis (set to 0.05), the VPD is the leaf to air vapor pressure deficit and C_S_ is the [CO_2_] at the leaf surface. The parameter g_1_ is here the target parameter to investigate plant internal water responses, as this parameter is interpretable as the inverse of water use efficiency and would only change upon soil or plant moisture stress (Medlyn et al. 2011; Zhou et al. 2013). The lower g_1_, the higher the water use efficiency and/or plant moisture stress.

In the following, we refer to the plant internal physiological gas exchange parameters J_max_ as the electron transport capacity and to g_1_ as the moisture stress parameter.

To fit model parameters, we used a global pattern search function on absolute residuals as the objective function followed by local iterative least squares regression with Huber weights using MATLAB (The MathWorks Inc., Natick, USA). Model fitting occurred on datasets that were split at noon for each of the three measurement days and for mycorrhizal and NM plants separately. The dataset comprised measurements of 75 to 80 leaves per day. This procedure provided parameter values of J_max_, α, Θ, R_D_ and g_1_ for each half day from sunrise to solar noon and from solar noon to sunset. The fittings were then used to interpolate rates for A_n_ and g_sw_ for non-observed time points, assuming that atmospheric conditions were identical for both treatments growing in the same glasshouse cabin. This resulted in continuous model interpolated daytime patterns of A_n_ and g_sw_ under ambient fluctuating conditions that represent the gas exchange of a mean canopy from the two uppermost fully expanded leaves of a random population of mycorrhizal and NM plants.

### Independent leaf traits and mycorrhizal colonization

Photosynthesis per leaf area measured in chambers of a particular size is influenced by leaf thickness or specific leaf weights, because different amounts of dry matter and, therefore, chloroplasts may be incorporated into the chamber. Therefore, we took pictures of three leaflets (one terminal leaflet and the first two oppositely arranged leaflets from the distal end) of the two uppermost fully expanded leaves, segmented the RGB picture to a binary picture with ImageJ (Schindelin et al. 2012), determined leaf area of the leaflets from calibrated pixel dimensions and calculated specific leaf weights (SLW) from the dried terminal leaflets (48 h, 60 °C). Tissue water fractions (TWF) were calculated from the difference of leaflet fresh and dry weights. Drying of whole plants occurred under the same drying conditions.

We measured leaf protein mass fractions (often called “concentrations”) as a surrogate for leaf N. Proteins formed with N are probably better functional predictors for photosynthetic potential on the leaf level than N mass fractions themselves and the determination of proteins required only sampling of the one or two leaflets which were also subject to gas exchange measurements. This was required to minimize destructive sampling of significant proportions of the leaf area which, if done, instantaneously changes the plant, e.g., root: leaf area ratios. Because N is remobilized and re-translocated in plants, a surrogate for leaf N of the leaf area/mass closely related to gas exchange assessments was desired.

The leaflets subject to gas exchange measurements were sampled and stored in liquid N_2_. Protein mass fractions from those leaflets were determined after Bradford (1976) with the extraction procedure published in Baxter et al. (2003). Briefly, 100 mg of frozen leaf tissue (N_2_) was ground with 1 mg of polyvinyl polypyrrolidine (PVPP) and 500 μL extraction buffer (50 mM HEPES-KOH, pH 7.5, 10 mM MgCl_2_, 1 mM EDTA, 2 mM DTT, 1 mM PMSF, 1 tablet of Pierce protease inhibitor per 50 samples, 0.1% Triton-X (*v*/*v*), 10% glycerol (*v*/*v*)). After thawing and centrifugation (12,000*g*, 4 °C, 2 min), desalting of the supernatant was done on a NAP 5 column (Sephadex G25 medium) and centrifuged through the column (1800 rpm, 3 min, 5810R Eppendorf, Hamburg, Germany). For protein determination via optical density at 595 nm, a standard curve of bovine serum albumin (1 mg mL^−1^) was used. Two microliters of the supernatant was mixed in 200 μL of Bradford reagent (Biorad, München, Germany) diluted with deionized water (1:5 *v*/*v*).

The sampling of leaflets occurred on the two uppermost fully expanded leaves of one plant. For SLW determination 20–24 terminal leaflets per day, treatment and plant were sampled (10–12 for both, the first and second uppermost fully expanded leaves). For protein mass fractions 12–14 opposite arranged leaflets were analyzed per day, treatment and plant (6–7 for both, the first and second uppermost fully expanded leaves). The detachment of 4 leaflets per plant constituted a reduction of only 1.5 to 2% of the total plant dry matter.

Fungal staining was done with trypan blue modified after Koske and Gemma (1989) on a fine root subsample of approx. 2 g. The samples were stored in 15% ethanol, incubated for 20 min at 60 °C in 10% KOH, acidified for 2 min in 2 N HCl and stained in 0.05% trypan blue in lactic acid (20 min, 60 °C). The grid line intersect method was used to determine the percent of mycorrhizal colonization in 100 root pieces (Giovannetti and Mosse 1980).

### Statistical analyses

Tests for normal distribution (Kolmogorov-Smirnov-Lilliefors-Test), homogeneity of variances (Levene-Test), ANOVA, and Pearson correlations were done with the STATISTICA 12 software (Statsoft, Tulsa, USA). In case of violations of assumptions after applying conventional data transformations, the non-parametric Kruskal-Wallis procedure was applied to assess significant differences at a cutoff level of *α* = 0.05. For differences in model parameters, non-overlapping confidence intervals (95%) were considered significant. For correlations of photosynthetic parameters with independent measured leaf traits (SLW, TWF, proteins), mean values of those leaf traits per plant and daytime were used.

## Results

The purpose of this study is to gain insight into the photosynthetic reaction of plants to inoculation with AMF, which is characterized by inherent variability and transiency. We hypothesized that this variability at least partially derives from different dynamics or realizations of plant internal traits and interaction with atmospheric conditions.

### Mycorrhizal colonization and plant growth

**Fig. 2:**
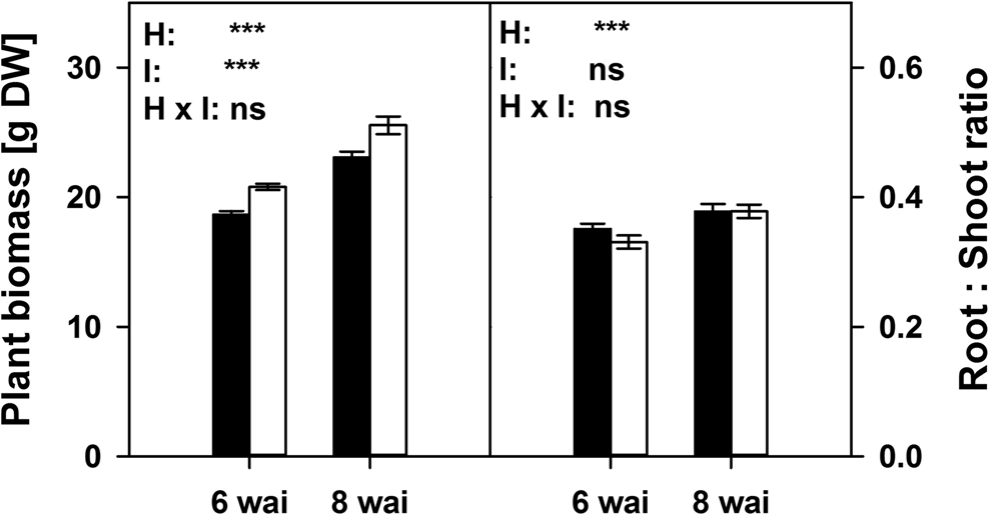
Plant biomass and root: shoot ratios of non-mycorrhizal (NM; black) and mycorrhizal plants (AM; white) harvested after the sixth and eighth week after inoculation (wai) with *F. mosseae*. Asterisks indicate significant differences (*P* < 0.001) of the factors harvest time (H), inoculation (I) and their interaction (two way ANOVA, *N* ≥ 20; ± se; *α* = 0.05)

Plants inoculated with AMF often show growth responses, which we also observed (Fig. 2). All mycorrhizal plants revealed a good colonization of root length by AMF structures (55% ± 2.96 se); NM roots were free of mycorrhiza. Mycorrhizal plants developed a mean 20.8 g of dry biomass; NM plants developed 18.7 g after 6 weeks of growth. After 8 weeks, mycorrhizal plants had 25.6 g of dry biomass and NM plants had developed 23.1 g. Within both harvests, the significant growth promotion by AMF constituted approx. 10%. Inoculation did not alter biomass partitioning between roots and shoots, but biomass partitioning shifted slightly towards roots from the sixth to the eighth week (Fig. 2). This occurred equally in mycorrhizal and NM plants.

### The leaf internal state during three arbitrary days under fluctuating growth conditions

We assumed that naturally fluctuating atmospheric conditions would create time dependent variation in leaf internal properties in plants of different sizes that are equally fertilized and irrigated in a constant pot size. We found that variability.

During the three arbitrary chosen days of gas exchange assessments, we observed treatment-induced differences in specific leaf dry weights (SLW) and tissue water fractions (TWF) in leaves. On day 1, mycorrhizal plants had higher SLW than NM plants (AMF, 3.5 mg cm^−2^; NM, 3.2 mg cm^−2^; *P* = 0.009, *N* ≥ 6) and lower TWF (*P* = 0.014, N ≥ 6). Those effects were absent on day 2 and 3 (Day 2: SLW, *P* = 0.280; TWF, *P* = 0.418; Day 3: SLW, *P* = 0.966; TWF, *P* = 0.732; N ≥ 6). This means that dry biomass and water contents per unit leaf area was variable between days and treatments.

To compare mycorrhizal effects on leaf photosynthetic capacity, we used the biochemical parameters of the photosynthesis and stomatal conductance models, which are interpretable irrespective of environmental conditions and are known to relate to plant internal traits that could possibly be influenced by AMF inoculation. These parameters were estimated separately for each half day and per treatment. We observed that mitochondrial respiration (R_D_^25^) was not affected by AMF (Table 1); hence, differences in net photosynthetic rates between treatments are at best marginally caused by changes in mitochondrial respiration rates. The electron transport capacity (J_max_^25^) as well as the moisture stress parameter (g_1_) showed variability among days and within phases of the day (Table 1). They were also comparatively higher, unchanged or lower in leaves of mycorrhizal plants during particular phases of the experiment (Table 1).

**Table 1:** Fitted gas exchange model parameters over days and treatments. The electron transport capacity for regeneration of ribulose bisphosphate at 25 °C leaf temperature (J_max_^25^), the mitochondrial respiration rate (R_D_^25^), and the water use efficiency parameter (g_1_) estimated at three different days in the morning (am) and afternoon (pm) during the sixth week of growth of plants inoculated with *F. mosseae* (AMF) and their non-mycorrhizal counterparts (NM). Values with non-overlapping confidence bounds were considered different between treatments and are highlighted in italics. On day 1, we could not reliably determine (n.d.) J_max_^25^, because of a lack of measurements of mycorrhizal plants under high light

			values		Confidence intervals [95%]		AMF effect
Parameter	Day	Daytime	NM	AMF	NM	AMF	
J_max_^25^ [μmol e^−^ m^−2^ s^−1^]	Day 1	am pm	*196* 141	***160*** n.d.	*194–198* 140–142	*158–162* n.d.	-
	Day 2	am pm	*126* 133	*148* 131	*124–127* 131–135	*146–149* 129–135	+ =
	Day 3	am pm	149 *145*	150 *201*	146–151 *143–148*	147–154 *194–209*	= +
R_D_^25^ [μmol CO_2_ m^−2^ s^−1^]	Day 1	am pm	1.25 1.44	1.27 1.54	1.17–1.33 1.38–1.51	1.22–1.31 1.46–1.62	= =
	Day 2	am pm	1.16 1.13	1.21 1.19	1.09–1.23 1.07–1.18	1.13–1.29 1.14–1.25	= =
	Day 3	am pm	1.23 1.08	1.25 1.13	1.13–1.33 0.99–1.16	1.19–1.33 1.04–1.22	= =
g_1_ [−]	Day 1	am pm	8.59 *9.67*	8.27 *7.33*	8.16–9.01 *9.36–9.98*	7.68–8.86 *6.98–7.68*	= -
	Day 2	am pm	*3.37* *2.02*	*2.68* *3.00*	*3.10–3.64* *1.87–2.17*	*2.48–2.88* *2.86–3.13*	- +
	Day 3	am pm	*5.65* 3.47	*9.15* 3.36	*5.35–5.95* 3.34–3.60	*8.55–9.74* 3.20–3.51	+ =

**Fig. 3:**
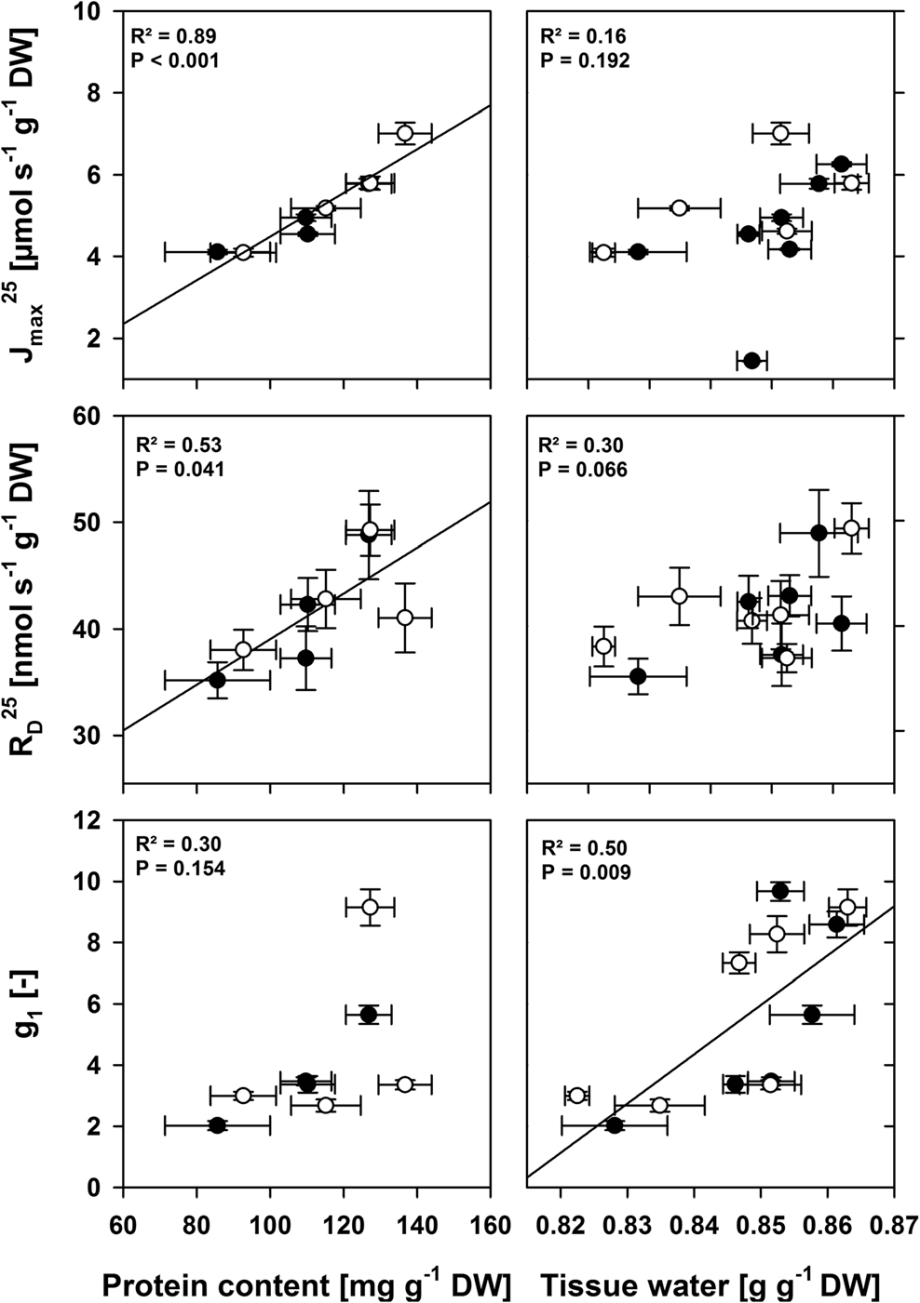
The electron transport capacity for regeneration of ribulose bisphosphate (J_max_^25^), the mitochondrial respiration rate (R_D_^25^) and the water use efficiency parameter (g_1_) in responses to leaf protein and tissue water fractions of plants inoculated with *F. mosseae* (AM, white) and their non-mycorrhizal counterparts (NM, black). Every data point represents a parameter estimated for one half day at the mean protein mass fraction or tissue water of the same leaves. The linear regression was carried out on all data combined (*N* = 12, horizontal errors: ± se, vertical errors: ± ci 95%, Protein mass fractions were not assessed on day 1: *N* = 8)

The parameters referring to unit leaf area may be influenced by changes to SLW as variable dry biomasses are introduced into the chamber with a distinct area. Hence, we scaled leaf protein mass fractions and all area-based parameters (J_max_^25^, R_D_^25^) to dry weight and related these to protein contents and tissue water of the leaves (Fig. 3). The results show that the processes largely depend on chloroplasts and leaf proteins, i.e., the electron transport capacity (J_max_^25^) and the mitochondrial respiration (R_D_^25^) are related to leaf protein mass fractions, but not to TWF. Since the parameters responded in similar ways to protein contents in mycorrhizal and NM plants, mycorrhizal plants may need to have enhanced protein contents per unit dry weight in order to show increased leaf electron transport capacities, possibly via improved nutrition of leaves. The opposite is the case for the moisture stress parameter g_1_, which only depended on TWF and could possibly be influenced by AMF via changes to the plant or soil hydraulic status. We are aware that tissue water contents may not be the most suitable leaf internal trait describing the plant hydraulic state or moisture stress, but in an independent experiment we observed that tissue water contents in tomato leaves scaled fairly well to leaf xylem water potentials (*r* = 0.72, *P* < 0.001, *N* = 48) and g_1_ was responding proportionally to xylem water potentials (*r* = 0.75, *P* < 0.001, *N* = 47). We used the TWF instead of the water potential here, simply to have a quick assessment of water relations within the measurement protocol, which would not have been possible in the required frequency with the pressure chamber method.

We found that electron transport capacity and the internal moisture state in mycorrhizal and NM plants were responding similarly to protein contents and TWF. This indicates that inoculation with AMF does not change the mechanistic basis of photosynthetic functioning.

### Simulation of CO_2_ assimilation responses to light intensities and vapor pressure deficits

**Fig. 4:**
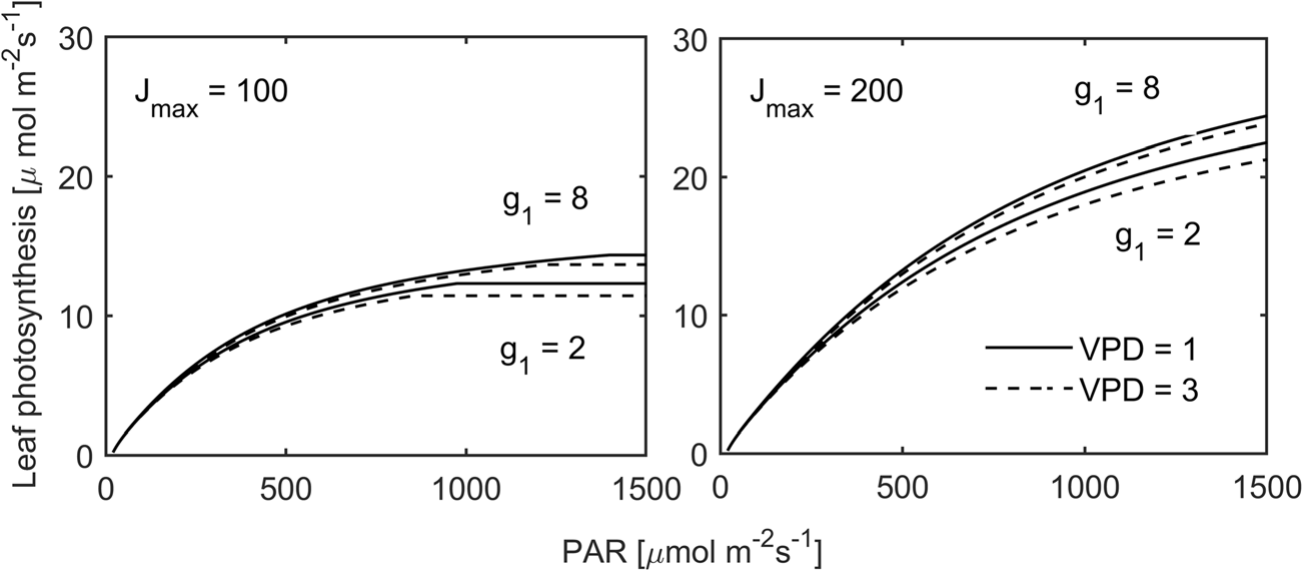
Model simulation of the light (PAR) response of leaf photosynthetic rates with the minimum and maximum electron transport capacities (J_max_) and internal moisture stress parameters (g_1_) observed during the experiment and for vapor pressure deficits (VPD) of 1 and 3 kPa

In order to illustrate how light (PAR) and atmospheric drought (VPD) would limit photosynthetic rates in leaves with a particular internal photosynthetic capacity, we simulated photosynthetic light response rates with parameter values of J_max_ and g_1_ resembling low and high values found in the experiment and with two levels of VPD (1 and 3 kPa). Figure 4 shows that under low light intensities VPD effects and plant internal moisture states (g_1_) are marginally changing photosynthetic rates, while electron transport capacity of leaves (J_max_) gradually allows higher photosynthesis with increasing PAR (Fig. 4). The internal moisture stress in leaves (g_1_) also induces the largest changes to photosynthetic rates under high light. If the plant moisture stress parameter g_1_ is high (low plant moisture stress), high atmospheric drought (VPD = 3 kPa) is less impactful in reducing photosynthesis than under higher plant moisture stress (g_1_ = 2).

### Mycorrhizal effects on the light response of photosynthesis

**Fig. 5:**
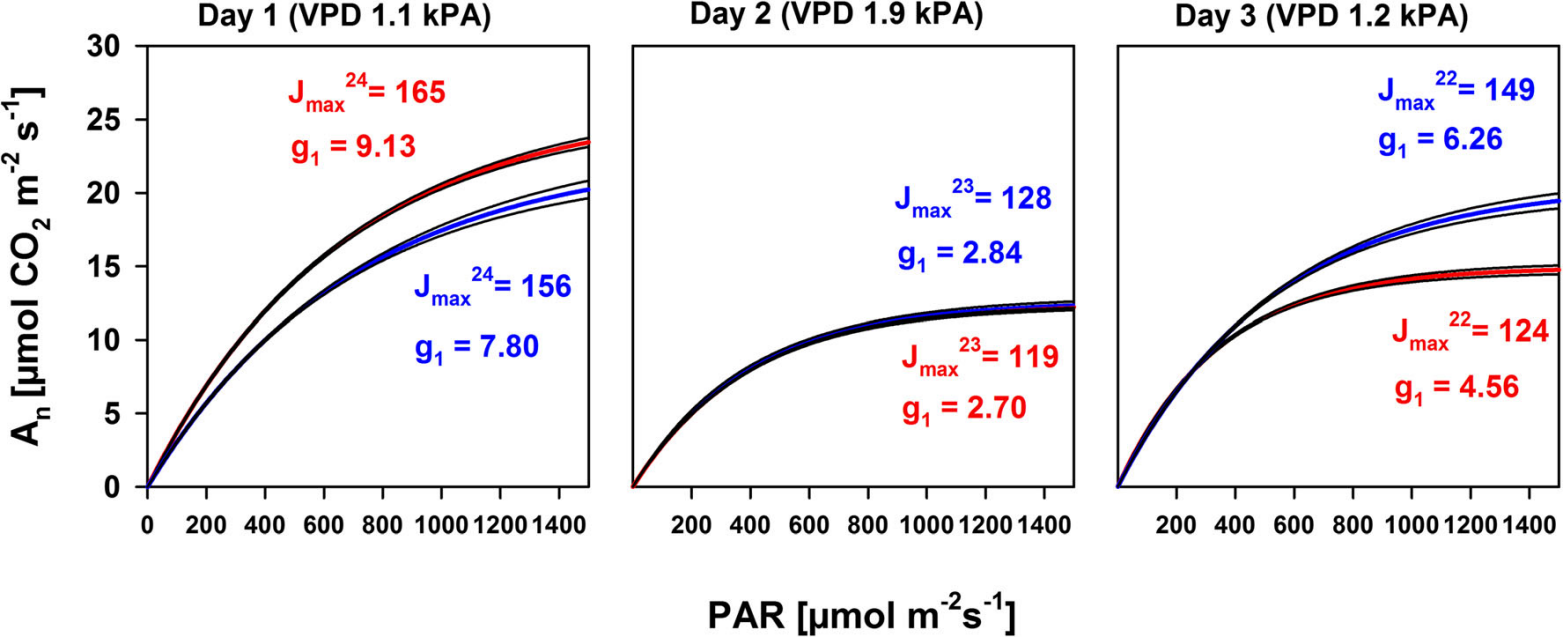
Photosynthetic rates (A_n_) in response to photosynthetic active radiation (PAR) of a mean canopy comprising the two uppermost fully expanded leaves of a population of plants inoculated (AM, blue lines) or not (NM, red lines) with *F. mosseae* based on three arbitrary days (1, 2, 3) within the sixth through eighth week of growth after inoculation. Black lines indicate the upper and lower 95% confidence limits of a two parameter exponential function fitted to data from measurements of 30 to 50 leaves per day and treatment. The mean daytime leaf to air vapor pressure deficit (VPD) is given and mean daytime model parameters are shown for AM and NM plants in the respective color. Parameters for J_max_ are presented as the actual electron transport rates assessed at the mean leaf temperature (T) present on the respective days (J_max_^T^)

In general, AMF inoculation did not or only marginally affected photosynthetic rates under low light conditions on any of the three assessment days, because photosynthesis is mainly light-limited (Fig. 5). At days with low to moderate atmospheric demands for water (Day 1 and 3), AMF inoculation induced changes to leaf photosynthetic rates under high light conditions, however, AMF did so negatively on day 1 and positively on day 3. Because photosynthesis under high light is mainly limited biochemically, e.g., by the electron transport capacity (J_max_) or by CO_2_ diffusion through stomata (g_1_), photosynthesis under high light conditions corresponded to the plant internal parameters determined in mycorrhizal and NM plants on the respective days. In contrast, on day 2, the day with the highest atmospheric demands for water, mycorrhizal effects on net photosynthetic rates under high light were absent. On day 2, both mycorrhizal and NM plants revealed the highest plant internal water stress (lowest g_1_). Positive effects of AMF on electron transport capacity (J_max_) did not become effective to increase high light photosynthesis, indicating that photosynthetic rates were strongly limited by stomatal closure, either by similar plant internal water stress (g_1_) and/or by the high VPD, which in turn limits maximum photosynthesis most strongly in water-stressed plants (see Fig. 4).

To this point, we have seen that plant internal photosynthetic capacities and plant internal moisture stress can vary between mycorrhizal and NM plants and between and even within phases of the day. In addition, we found indication that irradiance and the VPD set limits to photosynthetic rates that decide whether mycorrhizal effects on leaf properties become effective or not.

### The gas exchange response under fluctuating environmental conditions

**Fig. 6:**
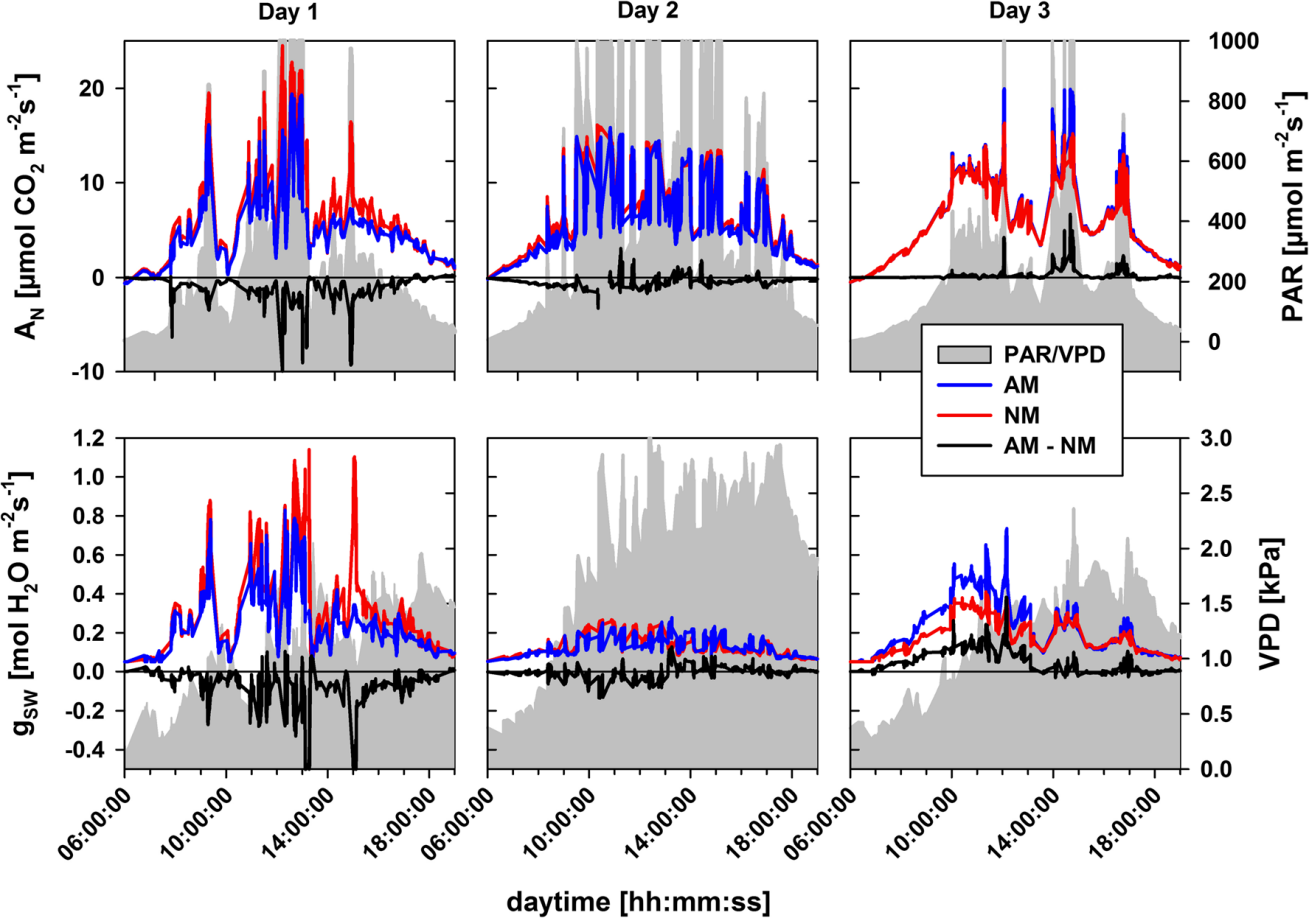
Daytime photosynthetic rates (A_n_) and stomatal conductance (g_sw_) of a mean canopy comprising the two uppermost fully expanded leaves of a population of plants inoculated (AM, blue lines) or not (NM, red lines) with *F. mosseae*. Black lines indicate the net difference between AM and NM canopies. The interpolated daytime patterns of three arbitrary days (1, 2, 3) within the sixth through eighth week after inoculation derived from measurements of 30 to 50 leaves per day and treatment. The gray areas refer to the secondary ordinate and display the patterns of photosynthetic active irradiance (PAR, top) and the leaf to air vapor pressure deficit (VPD, bottom) present in the glasshouse chamber assessed at the leaf surface

Short-term fluctuations in PAR and VPD are encountered in nature and open plant production systems. These fluctuations will determine whether photosynthesis is mainly limited by atmospheric or plant-internal factors and therefore, whether or not mycorrhizal effects on plant-internal states translate to changes in leaf photosynthesis. A mycorrhizal growth response depends on the supply of C from photosynthesis and, hence, also on absolute values of CO_2_ assimilation rates and the sum of events of mycorrhizal effects on photosynthesis. To get a deeper insight, we modeled time courses of actual photosynthesis and stomatal conductance of a representative sunlit canopy in response to instantaneous irradiance and VPD (Fig. 6).

At day 1, moderate atmospheric demands for water were present, with only occasionally occurring peaks in irradiance and the VPD remaining below 2 kPa. Compared to the other days, both NM and mycorrhizal plants had the lowest internal water stress and the highest electron transport capacities (see Fig. 5), which allowed on the one hand flexible stomatal conductance (g_sw_) and on the other hand the highest maximum rates of photosynthesis (A_n_) and g_sw_ under high light (Fig. 6). Mycorrhizal plants, however, were more water-stressed and had lower electron transport capacities than NM plants, consistent with lower tissue water contents as mentioned above. This led to high reductions in photosynthesis and stomatal conductance in mycorrhizal plants under high light. The differences in A_n_ between mycorrhizal and NM plants gradually declined with light intensities and were reduced to zero at PAR levels of 200 μmol m^−2^ s^−1^ (Fig. 6).

Day 2 was characterized by long phases of high irradiance and a VPD resembling a Mediterranean summer day. Here, g_sw_ was markedly reduced and showed greater upward damping in response to irradiance, clearly limiting A_n_ under high light intensities (Fig. 6, compare to day 1). No marked differences were observed for A_n_ and g_sw_ patterns between mycorrhizal and NM plants. This corresponded to low but similar tissue water contents in leaves of both treatments. Although the electron transport capacity in leaves (J_max_^25^) of mycorrhizal plants was higher in the first half day (see Table 1), this was not translating to higher A_n_ even under high light. Compared to the other days, the internal water stress was highest in mycorrhizal and NM plants, coinciding with the strongest atmospheric drought in the experiment (lowest g_1_ and lowest tissue water contents, see above). It indicates that mycorrhizal effects on plant internal traits were not translating to enhanced photosynthetic activity under high evaporative demands (high VPD) and/or strong plant drought stress.

On day 3, the day with the lowest atmospheric demands for water, the VPD rose during the day, which corresponded with increasingly reduced g_sw_ (Fig. 6). Although tissue water contents were equal in mycorrhizal and NM plants (*P* = 0.732, *N* ≥ 12), g_sw_ in mycorrhizal plants was higher with the largest changes during low VPD. This was coinciding with enhancements in g_1_, i.e., lower plant water stress. However, enhanced g_sw_ was only translating to increases in A_n_ under high light intensities. During that day, leaf protein mass fractions were significantly higher in leaves of mycorrhizal plants (*P* = 0.034, *N* ≥ 8). Within the second half day, g_1_ was similar in leaves of mycorrhizal and NM plants and differences in g_sw_ reduced, but A_n_ under high light still was higher in mycorrhizal plants, coinciding with improvements in J_max_ and protein mass fractions. The daily net carbon gain in a sunlit canopy of a population of mycorrhizal plants was low at approx. 1.4 mg C per m^2^, because of the short phases of high irradiance on that day. Nevertheless, those findings are similar to those on day 1, but in opposite directions. Electron transport capacity and plant moisture stress were translating to enhanced photosynthetic rates under high light conditions and comparable moderate atmospheric moisture (VPD).

To summarize, mycorrhizal and NM plants showed similar responses of electron transport capacities (J_max_) and internal moisture stress state (g_1_) to protein mass fractions and tissue water in leaves, respectively. But proteins, tissue water and related parameters differed during the phases of the experiment and between NM and mycorrhizal plants. Treatment differences translated to changes in photosynthetic rates only under high light and moderate atmospheric drought that allowed flexible regulation of stomata.

## Discussion

We investigated how leaf-internal properties that change in mycorrhizal plants are inducing differences to photosynthetic activity under variable climatic conditions. We showed that light conditions and atmospheric drought superimpose restrictions on the efficiency of leaf-internal properties and, thus, set a limit for photosynthetic activity, irrespective of whether plants are colonized by AMF or not. These atmospheric restrictions of photosynthetic activity can be beneficial for the resource efficiency of photosynthesis in mycorrhizal plants, because rates of photosynthesis remain equal even though mycorrhizal plants may experience stronger drought stress or have fewer proteins in leaf tissues than NM plants. Vice versa, the atmospheric limitations can be detrimental for resource efficiency of photosynthesis in mycorrhizal plants, because although these plants may be less water-stressed and may also contain more proteins in tissues than NM plants, nutrient-, and water-related benefits in mycorrhizal plants will not translate to higher carbon gain in leaves, i.e., higher photosynthetic rates. In either case, nutritional and water-related benefits of mycorrhizal plants become “superfluous” for photosynthetic activity and short-term carbon gain. This is the most important finding of this study and contributes to overcoming the stated limited predictability of mycorrhizal effects on leaf gas exchange (Augé et al. 2014).

### Mycorrhizal effects on leaf-internal photosynthetic potentials

Electron transport capacities, carboxylation capacity in the Calvin Cycle and triose-phosphate use underlie close homeostasis and determine maximum photosynthetic rates under light and/or CO_2_ saturation (Von Caemmerer 2000). These different biochemical capacities and, hence, light saturated photosynthetic rates can be influenced by AMF, because they depend on leaf [N], [P] (Braune et al. 2009; Niinemets et al. 2005; Walker et al. 2014) and protein mass fractions (Onoda et al. 2005; Yamori et al. 2010). Depending on source and amount of available nutrients, elevated leaf P mass fractions are frequent phenomena in mycorrhizal plants (Augé 2001; Ngwene et al. 2010, 2013; Nouri et al. 2014), but N mass fractions in leaf tissues have been variable, being higher, unchanged or lower depending on the setting (Augé 2001; Grimoldi et al. 2006; Leigh et al. 2009; Ngwene et al. 2013; Nouri et al. 2014). Thus, the biochemical model parameters closely related to nutrition such as J_max_ change in mycorrhizal plants (Adolfsson et al. 2015; Fini et al. 2011; Romero-Munar et al. 2017). Consistently, we found that high light photosynthetic rates were comparatively decreased or increased in mycorrhizal plants in accordance with variation in protein mass fractions and electron transport capacities (J_max_). The close relationship we found between protein mass fractions and J_max_ (see Fig. 3) indicates that N nutrition also is likely an important factor for carbon fixation in mycorrhizal systems. In this study, we did not aim to directly elucidate whether effects of AMF on leaf N-P stoichiometry (Augé et al. 2016) or sink stimulation of triose-phosphate use (Kaschuk et al. 2012) were quantitatively contributing to changes in light saturated photosynthetic rates. Still, the close J_max_-protein relationship and the decent treatment-induced co-occurrence of changes in J_max_ and measured photosynthetic rates under high light (see Figs. 3 and 5) indicate that protein mass fractions served as an appropriate proxy for leaf-internal biochemical limitation of photosynthesis for both mycorrhizal and NM plants.

Similarly to the reported inconsistent results of AMF influences on leaf N levels across studies, we found indication also for time-dependent variability of mycorrhizal contributions to tissue N levels by means of protein mass fractions. This caused reductions of J_max_ and high light photosynthetic rates on one assessment day and enhancements on another assessment day. This is an important finding, because it shows that the time point of measurement within 2 weeks crucially determines the results and, hence, the conclusions drawn from a single assessment.

The observation that J_max_ and photosynthetic rates under high light were comparatively repressed in mycorrhizal plants on day 1 and stimulated on day 3 may partially relate to the nutrient dynamics in our hydroponic system and to the differences in plant size. We used a mix of sand and vermiculite as the substrate, in which P immobilization is low, and applied readily available nutrients with the nutrient solution that had a high N/P ratio. That guaranteed substantial AMF colonization and avoided large nutrient-related growth responses because nutrient delivery by extraradical hyphae will mainly occur from exploration of additional pore space and—unlikely in our system—only marginally from remobilization. We wanted to avoid large mycorrhizal growth responses to achieve comparable sets of plants in terms of leaf age and flowering, because photosynthesis in a leaf is also a function of whole plant source-sink relations (Paul and Foyer 2001) and leaf age (Suzuki et al. 1987). Comparable sets of plants were achieved, but still a positive growth promotion upon AMF colonization was observed. Differences in plant sizes are especially critical in pots, where water and nutrient delivery from the periphery is blocked by a finite pot size and increased demands of larger plants cannot be compensated by increasing the rooted zone. In such hydroponic systems nutrient acquisition, especially N acquisition by plants is substantially mass flow driven and thus depends on plant transpiration and the substrate water content (Chapman et al. 2012). Normally, more water flows through larger plants per unit time (Augé et al. 2014). Consequently, in pots containing larger plants, substrate water contents deplete faster which may also impede potential nutrient uptake, because decreasing water contents serve to concentrate dissolved ions and decrease diffusional fluxes towards roots (Chapman et al. 2012). Nutrient diffusion coefficients of N and P change with moisture (e.g., Bhadoria et al. 1991; Gahoonia et al. 1994), solute mass flow differs with transpiration (e.g., Matimati et al. 2013) and transpiration itself is adjusted to soil moisture (e.g., Bitterlich et al. 2018b; Tardieu and Simonneau 1998). Indeed, we found indication that irrigation to pot water capacity may not have been sufficient to conserve an equal plant hydraulic state and leaf transpiration rates during Day 1, reflected by decreased TWF, stomatal conductance and the moisture stress parameter g_1_ in the mycorrhizal plants which were larger than NM plants. This might also result from an additive effect of several days, as assessment Day 1 was preceded by days of high atmospheric water demands (see Fig. 1). The comparatively insufficient irrigation to mycorrhizal plants could either have hampered mass flow driven N acquisition by mycorrhizal plants if reduced stomatal conductance caused lower whole pot evapotranspiration. Alternatively, similar rates of N uptake simply diluted N within the larger biomass of mycorrhizal plants. The opposite was the case on Day 3 where AMF induced an alleviation of plant moisture stress (lower g_1_, higher stomatal conductance), which was preceded by days of low atmospheric water demands (see Fig. 1). That possibly enhanced mass flow driven nutrient acquisition around Day 3 which was led to the observed increases in protein mass fractions. To prove these theoretical considerations, one would need to manipulate fertilization and/or irrigation regimes under which mycorrhizal plants expectably overcome plant nutrient and/or water limitations. But, if the hypothesized circular reference between nutrient and water acquisition applies to our system, it could be explanatory for our finding that changes to the daily mean plant-internal moisture stress (g_1_) always coincided with the level of electron transport capacity (J_max_; see Fig. 5) which closely relates to leaf nutrient and protein mass fractions. J_max_ is also found to typically decrease during drought exposure (e.g., Flexas et al. 2002; Xu and Baldocchi 2003).

One could deem the equal application of water and nutrients to different sized plants a mistake in cultivation by us, but we want to state a caveat here. With our untargeted approach, we show that detrimental effects of AMF on plant-internal traits regarding leaf nutrition and water stress can occur rapidly within days, far before visual observability. Nevertheless, phrases such as “irrigation after demand”, “in excess”, “thrice per week” are commonly found in studies targeting other mycorrhizal effects than water relations.

### Atmospheric limitations to leaf photosynthesis

The inconsistent differences in leaf-internal traits we found between mycorrhizal and NM plants were useful to address our main purpose in an unbiased way. During the experiment, we had scenarios in which mycorrhizal plants were comparatively more, equally or less stressed than NM plants regarding their leaf-internal photosynthetic potential. Thus, we can conclude how atmospheric conditions would exert control on leaf photosynthetic activity over a range of stress responses induced by AMF.

Theoretically, nutrient-, water-, or sink-related mycorrhizal effects on plants could lead to constantly changing photsynthetic rates if photosynthesis is limited mainly plant-internally, which is not always the case in nature and open plant production systems. Naturally fluctuating environments often comprise conditions under which atmospheric factors can limit photosynthetic rates, e.g., low light or high atmospheric drought. Fluctuating environmental conditions may also cause short-term switches between atmospheric and plant-internal limitations to photosynthesis. For example, changes in irradiance are transmitted with the speed of light (Einstein 1912), but benefits in leaf nutrition of mycorrhizal plants and sink strength by means of colonization intensity are expectably fairly constant from one hour to the next (Augé et al. 2014) and are rather conserved over short-term changes in environmental conditions (Konvalinková et al. 2015). Furthermore, changes in stomatal conductance upon AMF colonization may occur within hours or minutes (Augé et al. 2014) or suddenly, because hydraulic signals for stomatal regulation from underground can be transmitted with the speed of sound (Christmann et al. 2007).

The best studied atmospheric factor for photosynthesis of mycorrhizal plants is light, probably because it is rather conveniently implementable in factorial experiments by shading and/or illumination (reviewed in: Konvalinková and Jansa 2016). Translation of enhanced electron transport capacities to higher CO_2_ assimilation rates requires that leaves are not energy (NADPH)-limited at a particular level of substrate (CO_2_) availability, i.e., at high light intensities (Von Caemmerer 2000). This, we observed for mycorrhizal and NM plants in equal measure. But high light conditions occurred occasionally in our uncontrolled glasshouse experiment. Under low light, photosynthetic rates are dominantly sensitive to light (Ögren and Evans 1993), which is the reason why similar photosynthetic rates were found in canopies of mycorrhizal and NM plants under low irradiance. This frequent scenario may constitute a carbon costly scenario for mycorrhizal plants because an equally efficient photosynthetic canopy may not compensate additional carbon sinks such as AMF (Řezáčová et al. 2018). This is a feasible assumption for plants of similar size if AMF do not reduce other plant carbon sinks, but also for larger mycorrhizal plants when carbon demands of AMF scale with fungal biomass, which in turn scales with plant size. There is indeed strong indication that low light or shading reduces mycorrhizal growth responses (as reviewed in: Konvalinková and Jansa 2016). We can conclude that high light intensities are required for C gain efficiency of nutrient-related factors in leaves of mycorrhizal plants. Photosynthetic rates in leaves of mycorrhizal and NM plants are rather unaffected under low light, irrespective of leaf composition and/or inoculation.

Although atmospheric moisture is an important trigger for stomatal regulation, it has been assessed only one time regarding its superimposition of limitations to stomatal conductance in mycorrhizal plants (Huang et al. 1985). Stomata sense atmospheric drought and close with increasing VPD (e.g., Ball et al. 1987; Medlyn et al. 2011; Merilo et al. 2018). It was interesting to see that at day 1 and day 3, where lower VPDs were recorded than on day 2, stomatal conductance was comparatively reduced or increased in leaves of mycorrhizal plants in accordance with the plant internal moisture stress we assessed with g_1_. In contrast, on day 2 comprising a mean VPD similar to those found during a Mediterranean summer day, stomatal conductance became severely reduced which abolished translation of beneficial effects of AMF on electron transport capacity to elevated photosynthetic rates. We found indication that enhanced stomatal conductance in mycorrhizal plants requires at least moderate humidity that allows flexible stomatal regulation. In contrast, strong atmospheric drought does restrict potential mycorrhizal effects on stomatal conductance. This is consistent with the only other study we found, which showed that positive mycorrhizal effects on stomatal conductance were abolished at VPD levels higher than 2.1 kPa (Huang et al. 1985).

Atmospheric and plant-internal limitations as influenced by AMF may also act antagonistically. This we also observed. CO_2_ diffusion through stomata imposes diffusive limitations to photosynthesis, the leaf nutritional status determines how photosynthesis is limited biochemically (Grassi and Magnani 2005) and irradiance determines whether or not photosynthesis is energy-limited. Naturally, if stomatal conductance allows high CO_2_ availability at the sites of carboxylation, but the process is energy-limited, carbon cannot be assimilated to a greater extent than electron transport allows. This is exactly what we demonstrate here. On days when stomatal conductance was affected by AMF, photosynthetic rates under low light remained largely unaffected, hence mycorrhizal plants had higher or lower water use efficiencies of carbon gain, but equal photosynthesis (see Fig. 6, day 3). When light conditions approached saturating levels, photosynthetic rates become increasingly sensitive to CO_2_ and electron transport capacities, which allowed treatment-induced changes to photosynthetic rates. We can conclude that mycorrhizal effects on the physiological drought state of plants and photosynthesis becomes effective under conditions of high light intensities and low atmospheric drought.

### Potential consequences for mycorrhizal growth responses

The crucial finding of our study is that atmospheric moisture acts in parallel to light intensity; both cap beneficial mycorrhizal effects on leaf photosynthetic capacities either deriving from modulation of leaf composition and/or plant-internal moisture. The hypothesis that atmospheric factors can offset the effectiveness of increases in photosynthetic capacities of mycorrhizal plants for C fixation was confirmed. Carbon demands by AMF have to be compensated by whole plant photosynthesis if the mycorrhizal symbiosis should not result in growth depression or substitution for other sink processes in plants that are important for growth. A feasible assumption is that a more efficient leaf area in assimilating CO_2_ is a beneficial scenario for mycorrhizal plants and a trigger to stimulate plant growth. Benefits of AMF colonization for plants in CO_2_ assimilation potential cannot be utilized under conditions where photosynthesis is limited mainly by atmospheric factors and so, that situation should be avoided. If the latter is the case, but mycorrhizal effects on leaf nutrition and drought stress alleviation are present, photosynthesis remains equal, but its use efficiencies of nutrients and water may decrease in mycorrhizal plants. That constitutes scenarios under which mycorrhizal plants may deplete nutrient and water at increased rates but show unchanged rates of carbon acquisition. Indeed, photosynthetic water use efficiencies in mycorrhizal plants have been lower, unchanged or higher across studies (see reviewed in: Augé 2001) and upon specific water treatments (e.g., Birhane et al. 2012). We show here for the first time, that this variability can also occur within 2 weeks and even within days in mycorrhizal and NM plants growing in the same setting, which is at least partially explainable by atmospheric factors.

We do not claim that mycorrhizal plants growing under low light or atmospheric drought will always negatively respond to inoculation with AMF, because a mycorrhizal growth response will be a function of the interplay of atmospheric conditions, plant activity, mycorrhizal functioning and genetic factors. However, the duration of atmospheric limitations to photosynthesis within a culture period is likely crucial for the carbon gain response in comparative AMF studies. Environmental variability can be an important factor to determine mycorrhizal growth outcomes, which comprise parasitism, commensalism and mutualism (Johnson et al. 1997; Johnson and Graham 2013). Also, in terms of functioning, benefits for plants by mycorrhizal colonization with ecological significance may always appear as soon as physiological processes take place, irrespective of a net response (Smith and Smith 2011, 2012, 2013). Such discussions on functioning and outcomes of mycorrhizal symbioses concentrate on genetic factors, biotic environmental interactions, soil born abiotic factors like pH, temperature, structure, nutrient availability, transport and sources, and largely on symbiotic C-N-P relations. However, except for light, other atmospheric factors and drought are at best under-represented in those discussions, although nutrient availability, transport and C fixation integrate with water availability and flows from the soil to the atmosphere. The present study indicates that it may be worth also paying attention to water relations in the future when nutrient exchange budgets are investigated in mycorrhizal systems, because soil and atmospheric drought will affect symbiotic C-N-P trade-offs. We agree with other scientists working on carbon flows in AMF symbioses (Řezáčová et al. 2018) that integrative, plant-based and process-oriented approaches need to be used that account for environmental variability, to better understand mycorrhizal growth responses.
